# 2-*S*-Lipoylcaffeic Acid, a Natural Product-Based Entry to Tyrosinase Inhibition via Catechol Manipulation

**DOI:** 10.3390/biomimetics2030015

**Published:** 2017-08-10

**Authors:** Raffaella Micillo, Valeria Pistorio, Elio Pizzo, Lucia Panzella, Alessandra Napolitano, Marco d’Ischia

**Affiliations:** 1 Department of Chemical Sciences, University of Naples “Federico II”, Via Cintia 4, I-80126 Naples, Italy; raffaella.micillo@unina.it (R.M.); alesnapo@unina.it (A.N.); dischia@unina.it (M.d.I.); 2 Department of Biology, University of Naples “Federico II”, Via Cintia 4, I-80126 Naples, Italy; valeria.pistorio@gmail.com (V.P.); elipizzo@unina.it (E.P.)

**Keywords:** depigmenting agents, l-DOPA, dopachrome, tyrosinase, melanin, caffeic acid, dihydrolipoic acid, lipoic acid, keratinocytes

## Abstract

Conjugation of naturally occurring catecholic compounds with thiols is a versatile and facile entry to a broad range of bioinspired multifunctional compounds for diverse applications in biomedicine and materials science. We report herein the inhibition properties of the caffeic acid- dihydrolipoic acid *S*-conjugate, 2-*S*-lipoylcaffeic acid (LC), on mushroom tyrosinase. Half maximum inhibitory concentration (IC_50_) values of 3.22 ± 0.02 and 2.0 ± 0.1 µM were determined for the catecholase and cresolase activity of the enzyme, respectively, indicating a greater efficiency of LC compared to the parent caffeic acid and the standard inhibitor kojic acid. Analysis of the Lineweaver–Burk plot suggested a mixed-type inhibition mechanism. LC proved to be non-toxic on human keratinocytes (HaCaT) at concentrations up to 30 µM. These results would point to LC as a novel prototype of melanogenesis regulators for the treatment of pigmentary disorders.

## 1. Introduction

Several pigmentary disorders, such as melasma or lentigo, are associated with the overproduction or accumulation of melanin as the result of inflammatory responses or abnormal function of melanocytes inducing a local excess of pigmentation known as “hypermelanosis” [1,2,3]. The medical and aesthetical unfavorable impact of such disorders has prompted a constant search for new non-toxic depigmenting agents [4,5,6].

Since skin complexion is under control of several factors, including activity, expression, and stability of tyrosinase and related enzymes, melanocytes homeostasis, and melanosome transfer to the keratinocytes, commercially available depigmenting agents and melanogenesis regulators usually act through different mechanisms [7,8].

One of the most common approaches for control of pigmentation involves the inhibition of tyrosinase (EC 1.14.18.1) [9,10], a copper-containing enzyme exhibiting cresolasic or monophenolasic activity (hydroxylation of monophenols to *o*-diphenols) and catecholasic or diphenolasic activity (dehydrogenation of catechols to *o*-quinones) by a mechanism involving electron exchange with the copper atoms. In particular, tyrosinase catalyzes the key steps of melanogenesis, namely the hydroxylation and oxidation of l-tyrosine to dopaquinone [11,12].

When considering a new tyrosinase inhibitor several factors should be taken into account, such as product efficacy, cytotoxicity, solubility, cutaneous absorption, and stability. Increasing attention has been paid to the adverse effects of depigmenting agents in vitro and in vivo, especially further to the case of rhododendrol, a phenolic skin whitening agent that has been recently withdrawn from the market because of its cytotoxic effects on melanocytes and the consequent induction of leukoderma [13,14,15,16]. This has been ascribed to the tyrosinase-catalyzed oxidation of rhododendrol producing toxic metabolites [14,15,16,17].

Several catecholic compounds have raised interest due to their tyrosinase inhibition properties [18,19,20,21]. Among the catecholic compounds of natural origin, a prominent position is occupied by caffeic acid (3,4-dihydroxycinnamic acid) due to its health-beneficial properties [22,23,24]. A number of caffeic acid derivatives, mostly amides, have been described as tyrosinase inhibitors, while caffeic acid is not, and this ability has been attributed to both the structural similarity of the caffeic acid moiety to the substrate 3,4-dihydroxy-l-phenylalanine (l-DOPA) [20] and to the hydrophobicity and copper-chelating properties imparted by the particular nitrogen substituents [25,26,27,28].

Recently, with a view to synthesizing multifunctional antioxidants inspired to bioactive thiol-conjugates of naturally occurring phenolic compounds [29,30,31,32,33,34,35], we have focused our attention to dihydrolipoic acid (DHLA), the reduced form of lipoic acid (LA). The LA/DHLA system is known to be a powerful antioxidant being able to reduce reactive oxygen species, scavenge hydroxyl radicals, hypochlorous acid, and peroxynitrite, and chelate Fe^2+^ ions; moreover, LA and its reduced form can exert their functions both in membrane and in cytoplasm, because of their solubility in fats and water [36,37]. DHLA has also been reported to react with dopaquinone to give lipoyl-DOPA conjugates, and such a kind of reaction is able to affect melanin production resulting in depigmentation [38,39].

**Figure 1:**
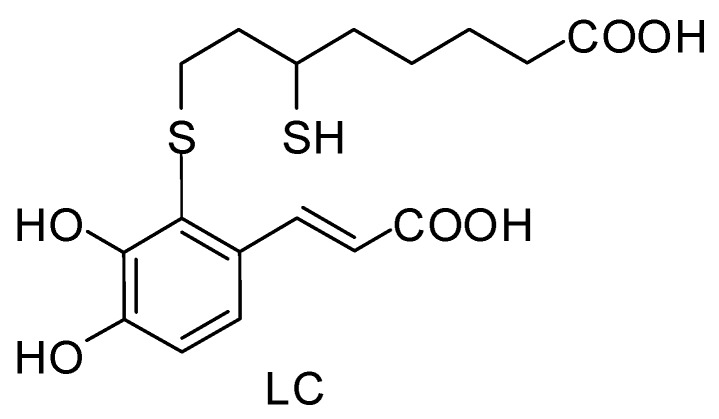
Structure of 2-*S*-lipoylcaffeic acid (LC).

On this basis, we report herein the tyrosinase inhibition properties of a conjugate of DHLA with caffeic acid, namely 2-*S*-lipoylcaffeic acid (LC) (Figure 1).

## 2. Materials and Methods

### 2.1. Materials

2-Iodobenzoic acid, oxone^®^, (±)-LA, sodium borohydride, sodium dithionite, l-DOPA, mushroom tyrosinase (EC 1.14.18.1), *p*-coumaric acid, caffeic acid, kojic acid, and 3-(4,5-dimethyl-2-thiazolyl)-2,5-diphenyl-2H-tetrazolium bromide (MTT) were purchased from Sigma-Aldrich (Milan, Italy). Dulbecco’s modified Eagle medium (DMEM), l-glutamine, penicillin/streptomycin, and fetal bovine serum (FBS) were purchased from Euroclone (Milan, Italy). All solvents were high-performance liquid chromatography (HPLC) grade. Double-distilled deionized water was used throughout the study.

2-Iodoxybenzoic acid (IBX) [40] and DHLA [41] were synthetized as reported.

### 2.2. Methods

Ultraviolet–visible (UV–Vis) spectra were recorded on a Jasco V-730 spectrophotometer (Lecco, Italy).

Nuclear magnetic resonance (NMR) spectra were recorded at 400 MHz on a Bruker instrument (Milan, Italy).

HPLC analyses were performed on an Agilent 1100 binary pump instrument (Agilent Technologies, Milan, Italy) equipped with a UV–Vis detector, using an octadecylsilane-coated column, 250 mm × 4.6 mm, 5 µm particle size (Phenomenex SphereClone ODS, Bologna, Italy) at 0.7 mL/min, and the following gradient: 0.1% formic acid (eluent a)/methanol (eluent b): 40% b, 0–10 min; from 40 to 80% b, 10–47.5 min. The detection wavelength was set at 280 nm.

Liquid chromatography–mass spectrometry (LC–MS) analysis was performed on an HPLC 1100 VL series instrument (Agilent Technologies) with an electrospray ionization source in positive ion mode (ESI+). An Agilent Eclipse XDB-C18, 150 mm × 4.60 mm, 5 µm (Agilent Technologies) was used, with the same eluent used for the HPLC analysis at a flow rate of 0.4 mL/min. Mass spectra were registered under the following conditions: nebulizer pressure 50 psi; drying gas (nitrogen) flow 10 L/min, at 350 °C; and capillary voltage 4000 V.

### 2.3. Synthesis of 2-*S*-Lipoylcaffeic Acid

A solution of *p*-coumaric acid (215 mg, 1.3 mmol) in methanol (18 mL) was treated with IBX (561 mg, 2 mmol) under vigorous stirring at room temperature. After 7 min a solution of DHLA (1.12 g, 5.3 mmol) in methanol (18 mL) was added dropwise, and after additional 15 min the reaction mixture was diluted with water and acidified to pH 1 with 6 M HCl. The mixture was then washed with hexane/toluene 8:2 *v*/*v* (10 × 200 mL) and extracted with chloroform (7 × 200 mL). The combined chloroform layers were dried over sodium sulfate and taken to dryness to afford pure LC (101 mg, 20% yield) as a yellow oil.

ESI+/MS: *m*/*z* 387 ([M + H]^+^), 409 ([M + Na]^+^); UV: λ_max_ (CH_3_OH) 252, 320 nm; ^1^H-NMR (CD_3_OD): δ (ppm) 1.38 (m, 1H), 1.54 (m, 1H), 1.54 (m, 2H), 1.42 (m, 1H), 1.62 (m, 1H), 1.78 (m, 1H), 1.81 (m, 1H), 2.26 (m, 2H), 2.88 (m, 1H), 2.90 (m, 1H), 2.96 (m, 1H), 6.29 (d, *J* = 16 Hz, 1H), 6.86 (d, *J* = 8.4 Hz, 1H), 7.22 (d, *J* = 8.4 Hz, 1H), 8.40 (d, *J* = 16 Hz, 1H); ^13^C-NMR (CD_3_OD): δ (ppm) 25.4 (CH_2_), 27.3 (CH_2_), 34.1 (2 × CH_2_), 39.3 (CH_2_), 39.4 (CH_2_), 40.2 (CH), 117.2 (CH), 118.1 (CH), 119.9 (CH), 121.7 (C), 130.7 (C), 144.2 (CH), 147.7 (C), 147.8 (C), 168.2 (C), 174.8 (C).

### 2.4. Mushroom Tyrosinase Inhibition Assay

One hundred microliters of a methanolic solution of LC were incubated in 2 mL (0.001–1 mM final concentration) of 50 mM phosphate buffer (pH 6.8) at room temperature in the presence of mushroom tyrosinase (20 U/mL). After 10 min 20 μL of a 100 mM solution of l-DOPA or l-tyrosine in 0.6 M HCl (1 mM final concentration) were added and the course of the reaction was followed spectrophotometrically measuring the absorbance at 475 nm for 10 min at 2 min intervals. In control experiments the reaction was run in the absence of LC. When required, the assay was performed as described but by adding l-DOPA to the reaction mixture soon after the addition of LC (3 µM).

In separate experiments, the assay was run as above with LC at 250 μM, in the presence or absence of l-DOPA, and after 10 min the mixture was analyzed by HPLC.

### 2.5. Investigation of the Mechanism of Inhibition of Mushroom Tyrosinase Activity

The assay was run as above, using different concentrations of l-DOPA (0.125, 0.25, 0.5, 1, and 2 mM) and LC (0, 2, 3 and 5 μM). Data were elaborated to build the Lineweaver–Burk plot.

### 2.6. Cell Viability Assay

Cytotoxic effects on immortalized human keratinocytes (HaCaT) were determined using the cell proliferation reagent MTT. Briefly, 5 × 10^3^ cells were seeded into a 96-well plate and were incubated overnight at 37 °C with 5% CO_2_. Medium was then replaced with 100 μL of fresh media containing LC at 0–30 µM and cells were incubated at 37 °C with 5% CO_2_. After 24, 48, or 72 h the LC-containing medium was removed, and 100 μL of fresh medium without red phenol, containing 10% MTT reagent, were added to each well and cells were incubated for 4 h at 37 °C in the dark. Subsequently, the absorbance at 570 nm was measured in a microtiter plate reader (SINERGY H4, BioTek, AHSI S.P.A., Milan, Italy) and cell viability was expressed as the mean ± standard deviation (SD) percentage compared to control.

## 3. Results and Discussion

### 3.1. Preparation of 2-*S*-Lipoylcaffeic Acid

The synthesis of LC was carried out by adapting a procedure previously reported for the preparation of the conjugation product of hydroxytyrosol with DHLA [30]. This involved the generation of the *o*-quinone of caffeic acid by the regioselective hydroxylation of *p*-coumaric acid with 2-iodoxybenzoic acid (IBX) [42], followed by addition of DHLA. The product was obtained in pure form in ca. 20% yield by a sequential extraction with solvents of increasing polarity, without the need for any chromatographic purification step. NMR, MS, and UV-Vis analysis confirmed the identity of the compound as the conjugation product of DHLA with caffeic acid quinone via the C-2 of the aromatic ring [43,44].

### 3.2. Inhibition of the Catecholase Activity of Mushroom Tyrosinase by 2-*S*-Lipoylcaffeic Acid

**Scheme 1:**
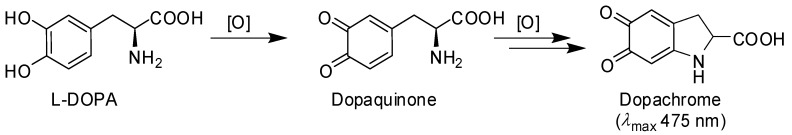
Dopachrome formation by tyrosinase-catalyzed oxidation of 3,4-dihydroxy-l-phenylalanine (l-DOPA). *λ*_max_: Wavelength of maximum absorbance.

The enzyme inhibition properties of LC were investigated using mushroom tyrosinase, which is routinely used for preliminary assessment of the activity of potential tyrosinase inhibitors [45,46,47]. For the assay of catecholase activity, l-DOPA was used as the substrate. The assay is based on the spectrophotometric monitoring of dopachrome formation (wavelength of maximum absorbance (*λ*_max_) 475 nm), following oxidative cyclization of dopaquinone produced by tyrosinase-induced oxidation of the substrate, in the presence and in the absence of the inhibitor [48] (Scheme 1).

**Figure 2:**
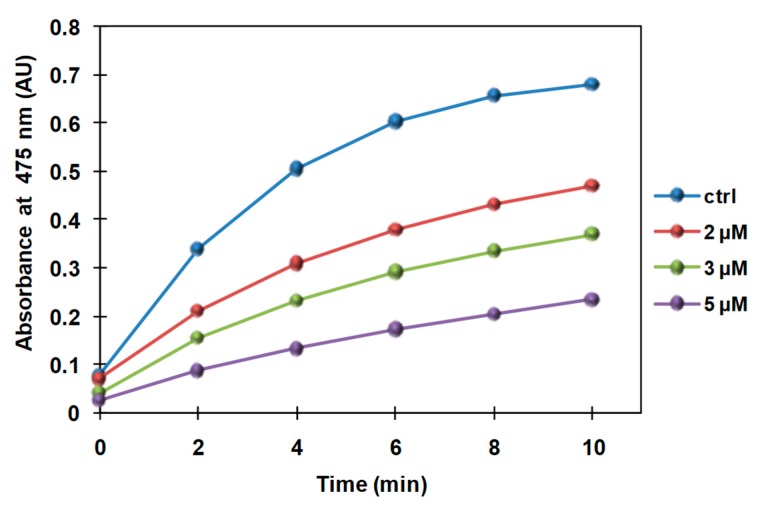
Time course of the absorbance change at 475 nm in the oxidation mixture of l-DOPA (1 mM) with mushroom tyrosinase in the absence (control (ctrl)) or presence of different concentrations of LC. Reported are the mean values of at least three experiments (standard deviation (SD) < 5%). AU: Arbitrary units.

LC was incubated in 50 mM phosphate buffer (pH 6.8) in the presence of mushroom tyrosinase (20 U/mL) at room temperature. After 10 min l-DOPA (1 mM final concentration) was added and the absorbance at 475 nm was measured at different times (Figure 2).

The percentage of inhibition was calculated using the following Equation:

(1) $$\%\;\mathrm{inhibition}=\left(1-\frac{{\Delta A}_{475/\min}\;\mathrm{in}\;\mathrm{the}\;\mathrm{presence}\;\mathrm{of}\;\mathrm{the}\;\mathrm{inhibitor}}{{\Delta A}_{475/\min}\;\mathrm{in}\;\mathrm{the}\;\mathrm{absence}\;\mathrm{of}\;\mathrm{the}\;\mathrm{inhibitor}}\right)\times 100$$

**Figure 3:**
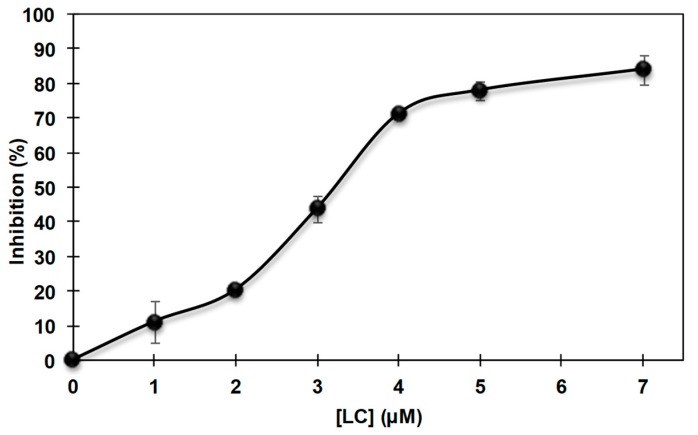
Percent of inhibition of mushroom tyrosinase activity vs. LC concentration using l-DOPA (1 mM) as substrate. Reported are the mean ± SD values of at least three experiments.

As reported in Figure 3, a maximum 80% inhibition was observed with 5 µM LC. A half maximum inhibitory concentration (IC_50_) value of 3.22 ± 0.02 µM was determined.

**Figure 4:**
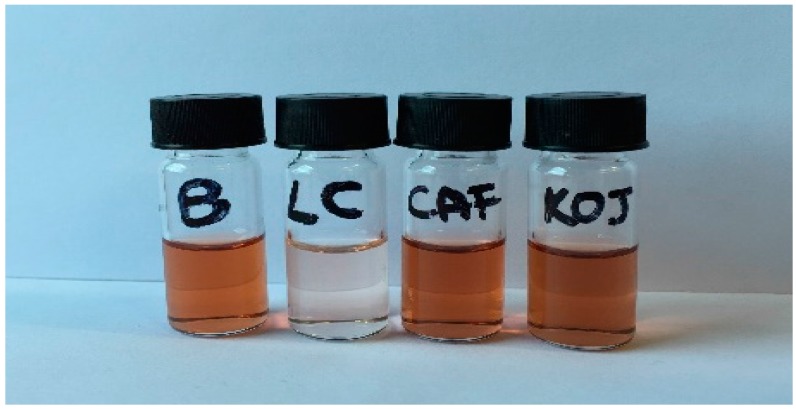
Tyrosinase-catalyzed oxidation mixtures of l-DOPA (1 mM) in the absence (B) or in the presence of 10 µM inhibitor (LC: 2-*S*-lipoylcaffeic acid; CAF: Caffeic acid; KOJ: Kojic acid).

Figure 4 shows the inhibition effect of 10 µM LC on the formation of dopachrome when l-DOPA is incubated with mushroom tyrosinase under the conditions previously described. Notably, at the same concentration, caffeic acid and the well-established tyrosinase inhibitor kojic acid [49,50] did not induce any inhibition.

These results not only show the superior inhibition properties of LC, but also underline the importance of the functionalization with DHLA in imparting tyrosinase inhibition properties to the parent catechol caffeic acid. It is well known that insertion of a chalcogen can affect the properties of catechol systems by lowering the O–H bond dissociation enthalpy [29,51,52]. However, the possibility that the higher inhibitory activity observed following conjugation with DHLA is not due to the presence of the sulfur substituent, per se, but rather to the hydrophobicity acquired by the compound cannot be excluded. Actually, the tyrosinase inhibition properties of caffeoyl-amino acidyl-hydroxamic acid derivatives have been ascribed in part to their hydrophobicity, making them suitable for binding to the active site of tyrosinase [26,27,28].

On the other hand, the primary sulfhydryl group of DHLA has been reported to react with dopaquinone produced by the tyrosinase-catalyzed oxidation of l-DOPA, leading to the formation of covalent lipoyl adducts and inhibiting dopachrome formation [38,39]. Given the presence of a free, although secondary, SH group, in separate experiments the possibility that LC could react with dopaquinone in the same way was investigated: the assay was run under the usual conditions and after 10 min the mixture was analyzed by HPLC which, however, did not reveal any consumption of the inhibitor nor formation of new products, ruling out a possible reaction of LC with the oxidation products of l-DOPA.

These results also suggested that LC is not an alternative substrate of the enzyme, since no consumption was observed even in the absence of l-DOPA. This is an issue of considerable importance, since the toxicity of some depigmenting agents has been mostly attributed to their acting as substrates of tyrosinase and, as such, being oxidized by the enzyme, leading to the formation of highly reactive, cytotoxic *o*-quinones [47,53,54].

Finally, the effect of pre-incubation on the inhibitory activity was investigated, by performing the spectrophotometric assay as above but with the addition of l-DOPA to the reaction mixture immediately after addition of LC. The time course of the absorbance change at 475 nm was comparable to that observed when LC was preincubated with the enzyme for 10 min before addition of l-DOPA, ruling out any role of pre-incubation in the inhibition effects exerted by LC.

### 3.3. Inhibition of the Cresolase Activity of Mushroom Tyrosinase by 2-*S*-Lipoylcaffeic Acid

**Figure 5:**
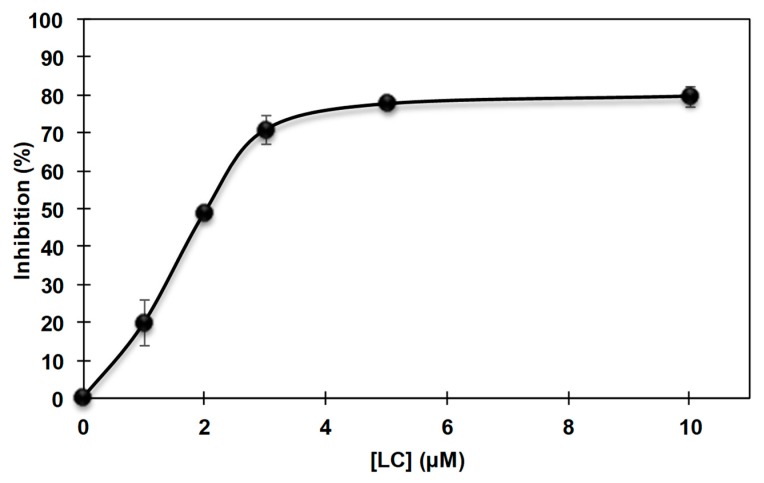
Percent of inhibition of mushroom tyrosinase activity vs. LC concentration using l-tyrosine (1 mM) as the substrate. Reported are the mean ± SD values of at least three experiments.

The ability of LC to inhibit the monophenolasic activity of mushroom tyrosinase was investigated as described above using l-tyrosine instead of l-DOPA as substrate (Figure 5). An IC_50_ value of 2.0 ± 0.1 µM was determined.

### 3.4. Investigation of the Mechanism of Inhibition of Mushroom Tyrosinase Activity by 2-*S*-Lipoylcaffeic Acid

**Figure 6:**
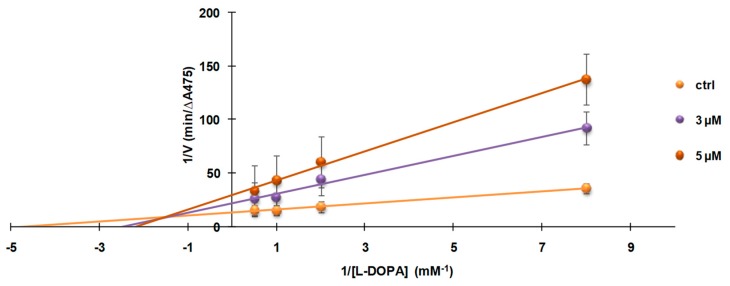
Lineweaver–Burk plot for the inhibition of mushroom tyrosinase-catalyzed l-DOPA oxidation by LC at 0 (control (ctrl)), 3 or 5 µM. Data were obtained as mean ± SD values of 1/V, inverse of the increase of absorbance at 475 nm per min (ΔA_475_/min), of three independent experiments with different concentrations of l-DOPA.

Lineweaver–Burk plot analysis was used to determine the mode of tyrosinase inhibition by LC. In Figure 6 the double-reciprocal plots of tyrosinase inhibition using l-DOPA as substrate is reported for different concentrations (0, 3, and 5 µM) of LC.

**Figure 7:**
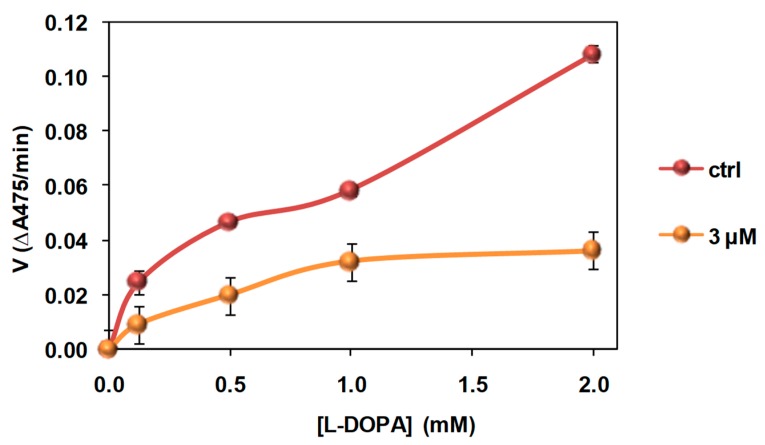
The effect of LC on the enzymatic kinetics for the mushroom tyrosinase-induced oxidation of l-DOPA. Data were obtained as mean ± SD values of the increase of absorbance at 475 nm per min (ΔA_475_/min) (V) of three independent experiments with different concentrations of l-DOPA.

The results were a family of straight lines with different slopes and different *x*- and *y*-intercepts, suggestive of a mixed inhibitor, lowering the maximum rate (*V_max_*) and increasing the Michaelis constant *K_m_* in a dose dependent-manner (Figure 7).

Several mixed-type inhibitors of mushroom tyrosinase have been described in the literature and, in most cases, complex kinetics are involved and the phenomena have been left unexplained. Recently, non-specific binding sites have been invoked to explain the mixed-type inhibition in mushroom tyrosinase activities [55]. However, in our case, available data do not allow discussion in more detail of how the ternary complex of substrate–enzyme–inhibitor is formed, to assess whether the free thiol group participates in the inhibition mechanism and by what mechanism, and what the role of the hydrophobic aliphatic chain of the DHLA residue is.

### 3.5. Cytotoxicity Evaluation

**Figure 8:**
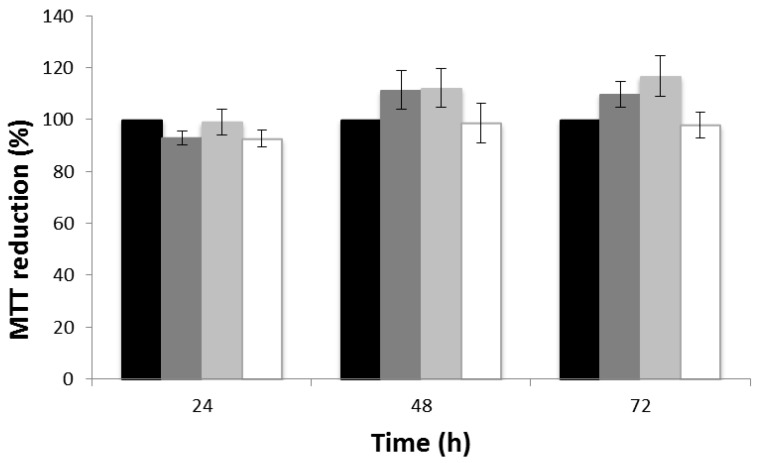
Effect of LC on HaCaT cell viability determined by 3-(4,5-dimethyl-2-thiazolyl)-2,5-diphenyl-2H-tetrazolium bromide (MTT) assay. Cells were cultured in normal growth medium and then subjected to treatment with LC (black: Control; dark grey: 0.3 µM; grey: 3 µM; white: 30 µM) for 24, 48, and 72 h. Cell viability was evaluated by measuring the A_570nm_. Results are expressed as the percentage (means ± SD from at least three experiments) compared to the control.

With the aim of evaluating the possible use of LC as a tyrosinase inhibitor in vivo, its cytotoxicity was preliminarily evaluated on human keratinocyte cells (HaCaT) by performing the MTT assay [52,56]. As shown in Figure 8, HaCat cells did not exhibit any significant reduction in proliferation rate when incubated with increasing amounts of LC over 72 h.

## 4. Conclusions

The use of natural catechols and derivatives as tyrosinase inhibitors for the treatment of pigmentary disorders associated with the overproduction or accumulation of melanin is well documented. We have reported herein that 2-*S*-lipoylcaffeic acid (LC), the *S*-conjugation product of caffeic acid and dihydrolipoic acid, is a promising lead structure for the development of catechol-based natural product-like tyrosinase inhibitors. LC was found to be able to inhibit both the catecholase and cresolase activity of mushroom tyrosinase with IC_50_ values as low as 3 µM, whereas under the same conditions caffeic acid did not show any effect, pointing to insertion of the dihydrolipoyl chain as an effective means of potentiating the inhibitory activity.

Whether this effect is due to the *S*-substituted catechol moiety alone or reflects the cooperative effect of the adjacent SH group cannot be assessed on the basis of the present data. It seems likely, however, that the potent inhibitory effects on tyrosinase reflect a synergic combination of caffeic acid as a substrate (l-DOPA)-like scaffold on which an efficient copper-binding arm (DHLA) is installed modulating catechol redox and chelating properties and conferring to the conjugate a higher degree of lipophilicity.

Although further experiments are needed to assess the actual potential of LC as a depigmenting agent in vivo, it is worth noting that the compound was non-toxic on immortalized human keratinocytes at concentrations up to 30 µM. Moreover, control experiments revealed that LC is not a substrate of tyrosinase, a critical issue for the toxicity of depigmenting agents in vivo. Although we are aware that additional, more cogent experiments are necessary to corroborate the lack of toxicity, the results of this paper can provide the necessary background for further studies on mammalian melanocyte cell lines, which will be directed to confirm activity on mammalian enzyme and pigment cells.

Overall, these results further expand the framework of the practical opportunities offered by the thiol-quinone coupling reactions, with particular reference to the dihydrolipoic/lipoic acid chemistry.
